# High Rates of Malaria among US Military Members Born in Malaria-Endemic Countries, 2002–2010

**DOI:** 10.3201/eid1709.110318

**Published:** 2011-09

**Authors:** Ellen R. Wertheimer, John F. Brundage, Mark M. Fukuda

**Affiliations:** Author affiliations: Armed Forces Health Surveillance Center, Silver Spring, Maryland, USA (E.R. Wertheimer, J.F. Brundage, M.M. Fukuda);; General Dynamics Information Technology, Fairfax, Virginia, USA (E.R. Wertheimer)

**Keywords:** Africa, malaria, emigrants and immigrants, epidemiology, prevention and control, military personnel, population surveillance, travel, United States, parasites, dispatch

## Abstract

To estimate malaria rates in association with birth country, we analyzed routine surveillance data for US military members. During 2002–2010, rates were 44× higher for those born in western Africa than for those born in the United States. Loss of natural immunity renders persons susceptible when visiting birth countries. Pretravel chemoprophylaxis should be emphasized.

Military members are at risk for malaria during assignments, deployments, and personal travel in malaria-endemic countries. They account for 5%–10% of all malaria cases reported in the United States. Immigrants from malaria-endemic countries are at risk for malaria when they visit their birth country (*1*). Visits to friends and relatives in malaria-endemic countries account for ≈50% of all malaria cases diagnosed in the United States.

Legal immigrants to the United States are eligible for military service, and many choose to serve. Malaria among US military members is tracked for health surveillance purposes by electronic records of medical encounters and notifiable medical events. These records are routinely transmitted to the Armed Forces Health Surveillance Center and integrated into the Defense Medical Surveillance System. To estimate rates of malaria in association with the birth countries of members of active components of the US military, we used these routine surveillance data for 2002–2010.

## The Study

For this report, a malaria case-patient was defined as a person hospitalized with a diagnosis of malaria (code 084 in International Classification of Diseases, 9th Revision, Clinical Modification) or reported as having a case of malaria through a military notifiable medical event reporting system. Persons could be considered malaria case-patients >1 time during the surveillance period but only 1 time during any 365-day period.

To determine locations where malaria infections were acquired, we used an algorithm that considered locations of malaria-related hospitalizations, travel histories reported on notifiable medical event records, and military assignment and overseas deployment records. The methods used by the Armed Forces Health Surveillance Center to identify malaria cases and their presumed locations of acquisition have been described in detail (*2*).

Countries of birth were self-reported during personnel security investigations (Entrance National Agency Checks) conducted among applicants for US military service. Rates of malaria by birth country were expressed as the number of malaria cases among all service members born in each country of interest per 10,000 person-years of active military service of all military members who were born in the respective countries.

During 2002–2010, a total of 835 malaria cases were reported among active military members; 5 persons were affected on 2 occasions at least 365 days apart. Compared with the overall composition of the US military, the proportions of military members with malaria were overrepresented by men (95%) and those 18–34 years of age (87%). Of all cases reported during the study period, 41%, 20%, and 3% were caused by *Plasmodium vivax*, *P. falciparum*, and other *Plasmodium* spp., respectively; *Plasmodium* spp. was not determined or reported for the other cases. The proportions of infections presumably acquired in Afghanistan, South Korea, and Africa were 42%, 21%, and 20%, respectively (data not shown).

Among military members affected by malaria during the study period, 624 (74.7%) were born in the United States, 107 (12.8%) were born in malaria-endemic countries other than Mexico, and 33 (4.0%) were born in countries where malaria is not endemic or in Mexico. The birth countries of the other 71 (8.5%) were not documented in available records. The most frequent known birth countries of case-patients were the United States, Nigeria (n = 24), and Ghana (n = 21). All other birth countries were represented by <8 cases each.

The highest malaria rates were among those born in Côte d’Ivoire (54.4/10,000 person-years), Togo (39.5/10,000 person-years), Cameroon (37.6/10,000 person-years), and Ghana (36.0/10,000 person-years). Of the 15 birth countries represented by at least 4 cases each, the 7 for which rates were highest were in western Africa. The malaria rate was 44× higher among service members born in 1 of the 7 western Africa countries (30.5/10,000 person-years) than among those born in the United States (0.70/10,000 person-years) (Figure).

**Figure:**
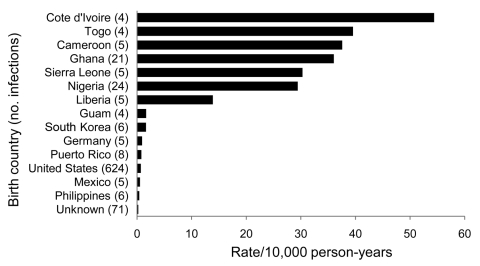
Rates of malaria infections (per 10,000 person-years of military service) by birth country, among birth countries represented by >4 malaria cases, active component military members, US Armed Forces, 2002–2010.

Among the 69 persons with malaria who were born in western Africa, 35 (50.7%) probably acquired the infection in their birth countries. In contrast, among the 38 malaria-infected persons born elsewhere, only 8 (21%) probably acquired the infection in their birth countries (Table).

**Table:** Numbers of malaria infections among 107 active component military members born in malaria-endemic countries, US Armed Forces, 2002–2010

Birthplace	Presumed location of malaria acquisition, no. infections		
	Birth country	Unknown	Other than birth country
Western Africa	35	27	7
Other malaria-endemic locations	8	2	28

Among the same 69 persons with malaria who were born in western Africa, the location of malaria acquisition was unknown for 27 (39%). Because these persons had no records of military assignments in or deployments to malaria-endemic areas, their infections were probably acquired during personal travel. If so, as many as 62 (90%) of these infections may have been acquired during visits to birth countries; in contrast, only ≈26% of infections of military members born in other malaria-endemic countries were potentially acquired in the respective birth countries.

## Conclusions

Among US military members, malaria rates are sharply higher among those born in western Africa than in other countries. Most malaria infections of those born in western Africa were probably acquired during visits to their birth countries.

The finding that immigrants to the United States have relatively high risk for malaria when they return to their malaria-endemic birth countries is not surprising (*3**–**5*). However, the finding that malaria rates among military members were 44× greater for those born in 7 western Africa countries than for those born in the United States requires attention.

Our findings should be interpreted with consideration of limitations. For example, cases were identified from diagnoses reported on administrative records of hospitalizations in US military and civilian (i.e., purchased care) medical facilities and from reports of notifiable medical events. Records of hospitalizations in deployed medical facilities (e.g., hospitals in the field, at sea) were not available; also, malaria diagnoses reported only on outpatient records were not considered cases. In turn, the cases enumerated here may underestimate the actual malaria infections that affected US military members during the study period. In addition, for 71 (8.5%) of the 835 military members with malaria, birth places could not be ascertained from records maintained for health surveillance purposes. The missing data may bias estimates of malaria rates in association with, as well as comparisons of rates across, birth countries. Finally, rate denominators (person-years of military service) do not account for the varying times that a person is at risk for malaria infection.

Before deploying to malaria-endemic areas, US military members are informed of the risks for and countermeasures against malaria (e.g., permethrin-impregnated uniforms and bed nets, DEET [N,N-diethyl-meta-toluamide]–containing mosquito repellent, chemoprophylactic drugs). Compliance with indicated countermeasures is mandatory, and enforcement is ensured by military supervisors. In contrast, before personal travel to these areas, counseling regarding malaria prevention may not be readily available or routinely accessed, and use of countermeasures is not enforced.

For persons residing in malaria-endemic countries, partial immunity develops in response to repeated exposures to malaria parasites. These persons may not be accustomed to or feel the need for chemoprophylactic drugs. However, after leaving their countries of origin, this acquired immunity wanes, leaving them susceptible to clinically significant malaria infection during subsequent visits to their birth countries. Because immigrants from malaria-endemic countries are at risk for malaria upon return to their birth countries, pretravel counseling of immigrants should emphasize the need for personal protective measures and encourage compliance with chemoprophylactic regimens before, during, and after the visits.
